# Cytotoxic Activity of* Holothuria leucospilota* Extract against* Leishmania infantum In Vitro*

**DOI:** 10.1155/2016/8195381

**Published:** 2016-02-28

**Authors:** Shahram Khademvatan, Alborz Eskandari, Jasem Saki, Masoud Foroutan-Rad

**Affiliations:** ^1^Research Institute for Infectious Diseases of Digestive System, Ahvaz Jundishapur University of Medical Sciences, Ahvaz, Iran; ^2^Department of Medical Parasitology and Mycology and Cellular and Molecular Research Center, Urmia University of Medical Sciences, Urmia, Iran; ^3^Department of Medical Parasitology, Faculty of Medicine, Ahvaz Jundishapur University of Medical Sciences, Ahvaz, Iran

## Abstract

Leishmaniasis is a tropical parasitic infection. The resistance and toxicity issues are the major complications and remain significant consequences related to the treatment of leishmaniasis with the recent and classical drugs. Thus there is an immediate requirement to develop new compounds for the treatment of this protozoan disease. Sea cucumbers or holothurians are potentially presented as the marine sources of antimicrobial and cytotoxic compounds. The aim of this study was investigation of* in vitro* antileishmanial activity of methanol extract of body wall, coelomic fluid, and cuvierian organs of* Holothuria leucospilota *obtained from coastal parts of Persian Gulf against* Leishmania infantum* promastigotes and axenic amastigotes. The colorimetric MTT assay was used to determine* L. infantum* promastigotes and axenic amastigotes viability at different concentrations of the extracts and drug control (Glucantime®) at time dependent manner and the results are represented as IC_50_ (50% of inhibitory concentration). Coelomic fluid was the most active extract among the three different extracts of* H. leucospilota* against* L. infantum *promastigotes and axenic amastigotes with IC_50_s of 62.33 *μ*g/mL and 22.4 *μ*g/mL and 73 *μ*g/mL and 46 *μ*g/mL at 48 and 72 hours after treatment, respectively. Cuvierian organs extract showed less toxicity with IC_50_s more than 1000 *μ*g/mL for both* Leishmania infantum* axenic amastigotes and promastigotes forms after 48 and 72 hours of exposure. Results acquired from the present study propose that the sea cucumber* H. leucospilota *may be a provoking source of antileishmanial compounds and could be a lead source in the development of the potent antileishmanial and cytotoxic drugs.

## 1. Introduction

Leishmaniasis is a protozoan parasitic infectious disease transmitted by the bite of certain phlebotomine sand flies and causing comprehensive morbidity and mortality in tropical and subtropical regions around the world, especially in most developing countries. It is caused by genus* Leishmania* and occurs in at least three major clinical manifestations: visceral, cutaneous, and mucocutaneous. The symptoms are ranging from self-healing ulcerative lesions to life threatening visceral leishmaniasis [1]. The occurrences of leishmaniasis could increase in the lack of an efficient vaccine and vector control problems and immunodeficiency caused by several conditions and the main current antileishmanial drugs administration limitations such as toxicity, resistance, parenteral administration, variable efficacy, and long schedules of their administration. Therefore, there is an immediate need for new compounds for the treatment of the leishmaniasis [2, 3].

Sea cucumbers or holothurians are marine invertebrate animals from the class Holothuroidea (echinoderms phylogenetically) which is located in the benthic places and deep seas all over the world [4]. They are utilized as human food sources, particularly in some districts of Asia. Because of the growing global need for food and medicine, many strains of sea cucumbers have been used [5]. These animals have a high percentage of protein with less cholesterol and are used as foodstuffs in various cuisines [6]. It has been reported in different studies that they have several bioactive compounds with biological activities. Antibacterial, antiviral, antioxidant, cytotoxicity, antitumor, and anticancer activities of the sea cucumber extracts and holothurians derived compounds have been reported previously [6]. It has been demonstrated that various protozoa and single-celled eukaryotes comprising* Leishmania *parasites display programmed cell death (PCD) mechanism similarities with the PCD of multicellular organisms exposed to certain cytotoxic agent [7]. Researchers demonstrated that sea cucumbers induce apoptotic cell death on various cancer cell lines [8–11]. Antileishmanial activity of these extracts and their bioactive compounds have not been studied so far particularly. This study was designed to investigate* in vitro* antileishmanial activity of various extracts of* H. leucospilota* against* L. infantum* promastigotes and axenic amastigotes in comparison to drug control Glucantime using colorimetric MTT assay.

## 2. Materials and Methods

### 2.1. Collection, Processing, and Extraction of Sea Cucumber

*H. leucospilota* samples were harvested freshly from the Persian Gulf, around the coastal cities of Qeshm and Kharg Islands, Iran. Samples were washed out and cleaned with fresh distilled water to detach detritus and external particles and stored at −20°C until the next usage in agreement with food and agriculture of the United Nations scale method [12]. Then the three parts of* H. leucospilota* (coelomic fluid, cuvierian organs, and body wall) were separated into several small pieces. Then, samples were kept at room temperature for two days to dry entirely and grinned into the fine powders. After that the different dried parts of* H. leucospilota* were extracted by maceration in appropriate quantities blending of methanol-water (50 : 50) and maintained for 16 hours. After that, the blend was filtered for two times. The filtrate was concentrated under vacuum conditions by rotary evaporation. Finally, to remove the solvents completely and increase the purity, all the extracts were dried by the freeze dryer. All other chemicals were purchased from Sigma (Sigma, Chemical Co., St. Louis, MO, USA).

### 2.2. Promastigote and Amastigote Culture

Standard strain of* L. infantum *(MCAN/IR/96/LONDON49) originally was supplied by Dr. Mohebali (Tehran University of Medical Sciences). Shortly, 5 × 10^5^ promastigotes/mL were cultured at 24 ± 2°C in RPMI 1640 medium with 10% of heat deactivated fetal calf serum (FCS), 2 mM L-glutamine, 100 mg/mL streptomycin, and 100 U/mL penicillin and after 96 hours promastigotes were subcultured at logarithmic phase and densities of 1 × 10^6^ cells/mL. Promastigotes were placed in 96-well culture plates at cell density of 1 × 10^5^ cells/100 *μ*L/well and treated with different concentrations of reference drug and* H. leucospilota* extracts were carried out in triplicate. When promastigotes grew up to logarithmic phase (1 × 10^6^ cells/mL), 100 *μ*L of suspension was transferred to a sterile falcon tube and 100 *μ*L of BHI medium with 10% of heat deactivated FCS and 1% of streptomycin and penicillin added to it. The promastigotes were centrifuged at stationary phase of growth and then supernatant was removed carefully and sediments were suspended in Schneider's medium (acidify with succinic acid) and incubated at 33 ± 2°C for 48 hours. The promastigotes were altered to amastigotes form and treated with different concentrations of reference drug and* H. leucospilota* extracts were carried out in triplicate.

### 2.3. Antileishmanial Assay

Colorimetric MTT assay was performed to determine antileishmanial activity of different extracts of sea cucumber and drug control. After the incubation periods at 24 ± 2°C for 24, 48, and 72 hours, 25 *μ*L of syringe filtrated MTT work solution (5 mg/mL) was added to wells and incubated at 24 ± 2°C for 4 hours. The supernatants were removed completely without interrupting the cell sediments, and 100 *μ*L of dimethyl sulfoxide (DMSO) was added to each well. After the microplate shaking for 15 min, the absorbance was recorded at 570 nm on a microplate reader. The viability of treated and untreated promastigotes was studied by measuring conversion of MTT to the reduced form that indicates cell viability, and a decline in the amount of MTT alteration is a toxicity indication to the promastigotes [13–15]. Relative numbers of viable cells are prescribed by the following equation:

(1) $$\begin{array}{c} Viable\;\;cells\left(\;\%\;\right)=\frac{\left(A_{T}-A_{B}\right)}{\left(A_{C}-A_{B}\right)}\times 100, \end{array}$$

where *A*_*T*_, *A*_*C*_, and *A*_*B*_ are the absorbance of treated, untreated, and blank samples, respectively, which were studied as means of triplicate. IC_50_ (concentration of drug decreasing 50% of cell viability) was prescribed by linear regression analysis [13]. Reference drug, Glucantime® (Meglumine antimoniate), was kept at 4°C and diluted with RPMI 1640 medium at the time of incubation. Sterile distilled water was used to prepare stock solutions of all extracts of* H. leucospilota* and twofold dilution method was used to prepare serial concentration of the extracts (156 × 10^3^–4.75 *μ*g/mL) to make working solutions.

### 2.4. Determination of Morphological Changes of Promastigotes

Promastigotes treated with* H. leucospilota *extracts (IC_50_) were tested to perceive morphological changes. Promastigotes were centrifuged for 10 min at 1000 g and supernatant was removed carefully and the sediments were suspended in PBS. Morphological alteration was studied after treatment under light microscope on the magnification of ×100 at different time points from 0 to 72 hours [13, 16].

### 2.5. Statistical Analysis

All the results were expressed as the mean values of three independent experiments ± SE by linear regression. The data were analyzed by SPSS 21.

## 3. Results

**Figure 1:**
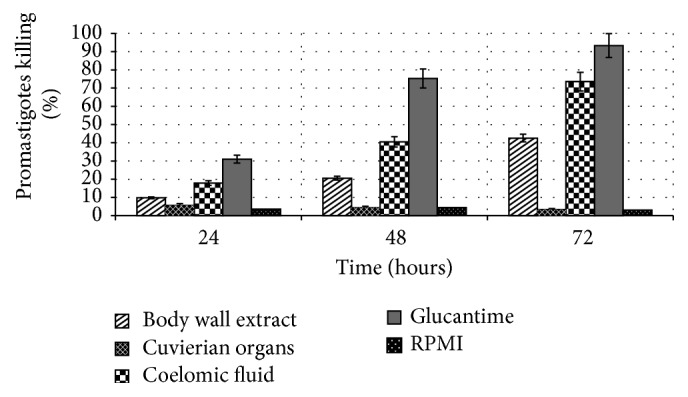
Time dependent leishmanicidal activity of* H. leucospilota* extracts against* L. infantum* promastigotes. The results are shown as a percentage of parasite killing from three independent experiments performed as triplicate (mean ± SE) using MTT assay after treatment with 50 *μ*g/mL of each sample tested.

**Figure 2:**
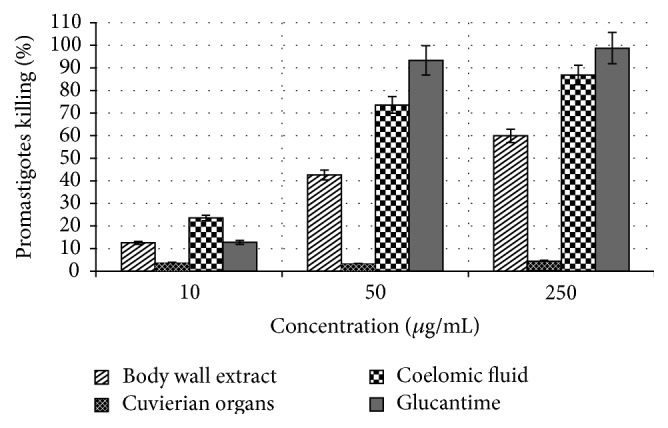
Concentration-dependent leishmanicidal activity of* H. leucospilota* extracts against* L. infantum *promastigotes. The results are shown as a percentage of parasite killing from three independent experiments performed as triplicate (mean ± SE) using MTT assay after 72 hours of exposure.

Methanol extract of* H. leucospilota* demonstrated significant leishmanicidal activity against* L. infantum* promastigotes* in vitro* at different concentrations and time dependent manner (Figures 1 and 2). The* in vitro* antileishmanial effects of* H. leucospilota* extracts and Glucantime against* L. infantum* promastigotes and amastigotes are shown in Table 1. These results revealed that of all sea cucumber extracts body wall and coelomic fluid of* H. leucospilota* have antileishmanial activity with IC_50_s of 53.5 *μ*g/mL and 22.41 *μ*g/mL after 72 hours of incubation, while cuvierian organs extracts have shown no significant leishmanicidal activity on extracellular form of* L. infantum*. Coelomic fluid extract of this sea cucumber was the most active extract against both extra- and intracellular forms of* L. infantum* with IC_50_s of 62.33 *μ*g/mL and 22.41 *μ*g/mL and 73.1 *μ*g/mL and 46.21 *μ*g/mL after 48 and 72 hours of exposure, respectively. Promastigotes survival for body wall as well as coelomic fluid extract at this concentration point was 90.24%, 79.4%, and 57.45% and 82.13%, 59.66%, and 26.43% after 24, 48, and 72 hours of treatment, respectively (Table 1 and Figure 1).

**Figure 3:**
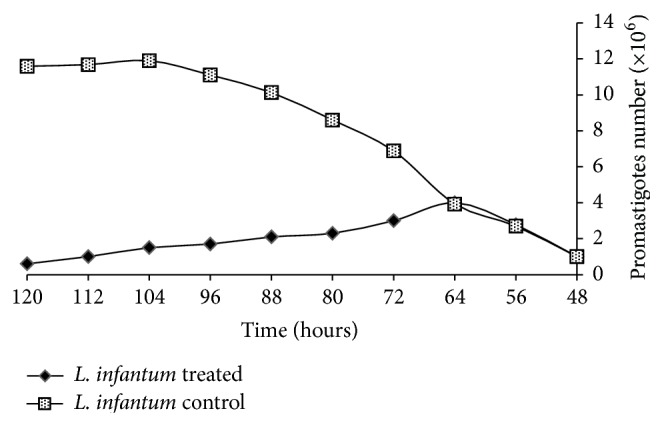
Antileishmanial activities of coelomic fluid extract of* H. leucospilota* against* L. infantum *promastigotes. Number of promastigotes treated with or without coelomic fluid (22.5 *μ*g/mL) at different time point after 48 h.

**Figure 4:**
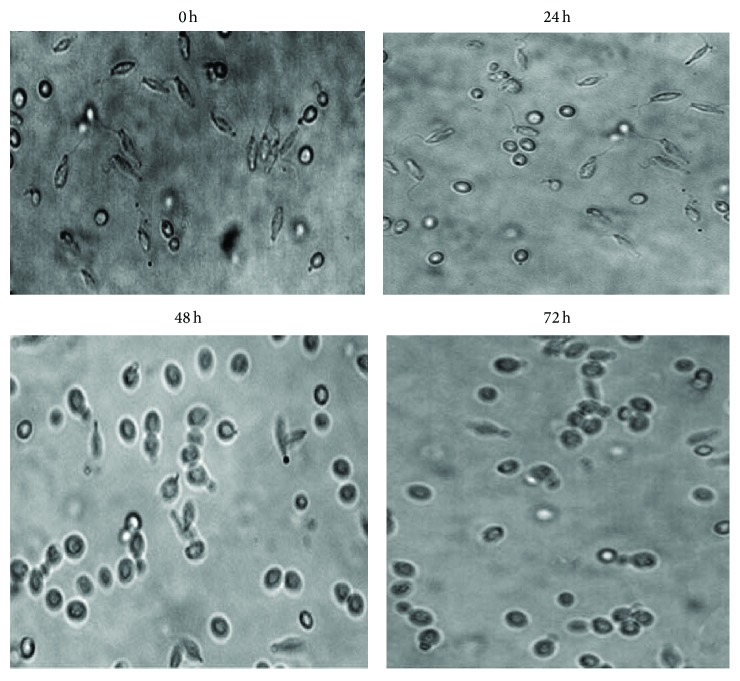
Morphological changes of* L. infantum *promastigotes treated with coelomic fluid extract (22.5 *μ*g/mL) at different time point after 24, 48, and 72 h.

The morphological alterations of treated promastigotes with or without 22.5 *μ*g/mL (IC_50_) of coelomic fluid of* H. leucospilota* were carried out in the logarithmic growth phase of* L. infantum* promastigotes and studied under a light microscope at 8 h intervals after coelomic fluid extract adding up to 72 hours. The number of promastigotes at the inception of treatment with coelomic extract was one million/mL approximately, and the treated promastigotes grew up to 4 million/mL, 16 hours after exposure, and the live promastigotes quantity decreased in 16 to 72 hours after exposure in comparison to untreated samples, which grew up to 12-13 million/mL up to 120 hours. Evaluation of the microscopic changes of promastigotes exhibited that promastigotes began to contract at 8 hours after treatment of* L. infantum* promastigotes (Figure 3). At the end of 72 hours of exposure, all the treated promastigotes compared with the control promastigotes exhibited cytoplasmic shrinkage and size diminishing (Figure 4).

## 4. Discussion

Nowadays pentavalent antimonial compounds are the most important drugs that are being used for treatment of leishmaniasis. The resistance and toxicity are the major complications and significant issues related to the treatment of leishmaniasis infection with these recent and classical drugs [2, 3].

Pharmacological activities such as antiproliferative, antioxidant, anti-inflammatory, anticoagulant, anticancer, antifungal, and antimicrobial activities of the bioactive compounds derived from benthic organisms, mainly from star-fish and sea cucumbers, have been examined in several studies previously [17–19]. Fatty acids of sea cucumber, containing arachidonic acid, docosahexaenoic acid, and eicosapentaenoic acid, appear to have a potential role in wound healing and tissue repairing [6]. For this reason, many parts of the world utilize sea cucumbers as a traditional treatment for burns and cuts [20, 21]. Furthermore, sea cucumber, due to possessing arginine, glycine, and glutamic amino acids ingredients, could contribute to the immunomodulatory activity [22]. Accordingly it is suggested that sea cucumber and its certain bioactive amino acid and fatty acid can improve immune system function [22] and lessen the duration of wound healing [21, 23]. It is suggested that certain molecules with anticancer activity at the pharmacological observation might be also suitable as antiprotozoal agents. Accordingly, several chemical and natural compounds have been examined against trypanosomatids and other single-celled parasites [3, 24]. Thus researchers indicated that sea cucumber can induce apoptotic cell death cytotoxicity effects on various cancer cell lines [8–11, 25]. Antifungal and antibacterial activities of sea cucumber* H. leucospilota* isolated from Persian Gulf have been studied recently in several studies, and they showed bactericidal and fungicidal effects [12]. Cytotoxic effect of this Persian Gulf sea cucumber extract against K-562 and WEHI-164 cancer cell lines has been examined, and results demonstrated that aqueous coelomic extracts of* H. leucospilota* have satisfactory levels of cytotoxicity effect against these two cancer cell lines [26].

In current research, the effectiveness of several concentrations of the* H. leucospilota* extract against* L. infantum* promastigotes as well as axenic amastigotes was examined. We have investigated the methanol extract of coelomic fluid of* H. leucospilota* that exhibited 73.56% inhibition of promastigotes after 72 hours of treatment with concentration of 50 *μ*g/mL compared with Glucantime with 93.34% inhibition. Among all the three* H. leucospilota* extracts, coelomic fluid was the most active extract against extra- and intracellular forms of* L. infantum* after 48 and 72 hours of treatment. Although Glucantime showed highest activity against* L. infantum* promastigotes and amastigotes, the cuvierian organs extract with IC_50_s > 1000 *μ*g/mL was the lowest active extract on these extra- and intracellular forms of this parasite after 48 and 72 hours of treatment, respectively. Body wall extract was less effective than coelomic fluid at 24, 48, and 72 hours and 3 times less effective than Glucantime after 24 and 48 hours after treatment. Furthermore time-dependency after 24, 48, and 72 hours of exposure of extracts and drugs control to* Leishmania* promastigotes, as well as concentration-dependency at three values of 10, 50, and 250 *μ*g/mL, was determined (Figures 1 and 2). These extracts reduced promastigotes viability at different concentrations in a concentration-dependent and time dependent manner.

## 5. Conclusion

This study revealed satisfactory level of* in vitro* activity of* H. leucospilota* against* Leishmania* parasite; however,* in vivo* leishmanicidal activities of this agent and its constituents should further be examined. Generally, leishmanicidal effect of extracts derived from this invertebrate holothurian may be due to the presence of bioactive components such as triterpene glycosides, concentrated mainly in the coelomic fluid in comparison with cuvierian organ with lesser activity. Finally, results obtained from this study propose that* H. leucospilota* could be sources of antiprotozoal compounds that could be used as a lead potent leishmanicidal agent.

## Figures and Tables

**Table 1:** *In vitro* antileishmanial activity of *H. leucospilota *and reference drug Glucantime against *L. infantum *promastigotes and amastigotes in different time points.

Test sample/drug	Promastigotes			Survival^b^ (%)	Amastigotes		
	IC_50_^a^ (*µ*g/mL)				IC_50_^a^ (*µ*g/mL)		
	24 h	48 h	72 h	50 *µ*g/mL	24 h	48 h	72 h
Body wall extract	674 ± 15	275.25 ± 5.4	53.5 ± 2.5	57.45 ± 3.2	521.11 ± 5	318.21 ± 2.2	74.2 ± 6.4
Cuvierian organs	>1000	>1000	>1000	>1000	>1000	>1000	>1000
Coelomic fluid	367 ± 8.4	62.33 ± 4.1	22.41 ± 1.5	26.43 ± 1.7	229 ± 6.5	73.1 ± 3.3	46.21 ± 1.2
Glucantime	75.5 ± 3.5	25 ± 2.2	12.65 ± 1.25	6.6 ± 5.4	96.32 ± 2.3	21.1 ± 4.3	7.9 ± 2.4

^a^Data are presented as the means ± SD of results from triplicate experiments.

^b^Survival of promastigotes after 72 hours of exposure to 50 *µ*g/mL concentration of test compounds.
